# Identification of a Putative Quantitative Trait Gene for Resistance to Obesity in Mice Using Transcriptome Analysis and Causal Inference Tests

**DOI:** 10.1371/journal.pone.0170652

**Published:** 2017-01-23

**Authors:** Akira Ishikawa

**Affiliations:** Laboratory of Animal Genetics, Graduate School of Bioagricultural Sciences, Nagoya University, Nagoya, Aichi, Japan; JAPAN

## Abstract

It is still challenging to identify causal genes governing obesity. *Pbwg1*.*5*, a quantitative trait locus (QTL) for resistance to obesity, was previously discovered from wild *Mus musculus castaneus* mice and was fine-mapped to a 2.1-Mb genomic region of mouse chromosome 2, where no known gene with an effect on white adipose tissue (WAT) has been reported. The aim of this study was to identify a strong candidate gene for *Pbwg1*.*5* by an integration approach of transcriptome analysis (RNA-sequencing followed by real-time PCR analysis) and the causal inference test (CIT), a statistical method to infer causal relationships between diplotypes, gene expression and trait values. Body weight, body composition and biochemical traits were measured in F_2_ mice obtained from an intercross between the C57BL/6JJcl strain and a congenic strain carrying *Pbwg1*.*5* on the C57BL/6JJcl background. The F_2_ mice showed significant diplotype differences in 12 traits including body weight, WAT weight and serum cholesterol/triglyceride levels. The transcriptome analysis revealed that *Ly75*, *Pla2r1*, *Fap* and *Gca* genes were differentially expressed in the liver and that *Fap*, *Ifih1* and *Grb14* were differentially expressed in WAT. However, CITs indicated statistical evidence that only the liver *Ly75* gene mediated between genotype and WAT. *Ly75* expression was negatively associated with WAT weight. The results suggested that *Ly75* is a putative quantitative trait gene for the obesity-resistant *Pbwg1*.*5* QTL discovered from the wild *M*. *m*. *castaneus* mouse. The finding provides a novel insight into a better understanding of the genetic basis for prevention of obesity.

## Introduction

Genome-wide quantitative trait locus (QTL) mapping is performed on the basis of either association analysis in outbred populations (called a genome-wide association study (GWAS)) or linkage analysis in three-generation pedigrees or designed crosses (a genome-wide QTL analysis). QTL mapping is an unbiased phenotype-driven, discovery-based approach without any priori information on candidate genes for a complex disease of interest, and it is used to identify chromosomal regions of disease QTLs and to understand the genetic basis and molecular basis of disease susceptibility. Until now, large numbers of QTLs and genetic variants for complex disease traits including obesity have been reported in humans and model animals, and they have been deposited in databases such as the NHGRI-EBI GWAS Catalog [1] and the Mouse Genome Database (MGD) [2]. However, it is still challenging to identify causal quantitative trait genes (QTGs) and causal quantitative trait nucleotides (QTNs) for so-called QTLs with relatively small phenotypic effects [3,4], though there have been several successful examples [4–7].

We previously revealed 24 QTLs with main effects and/or epistatic interaction effects on postnatal body weight and growth in mice by genome-wide QTL analyses with an intersubspecific backcross population between C57BL/6JJcl (B6), a commonly used inbred strain prone to obesity and type 2 diabetes [8], and wild *Mus musculus castaneus* mice trapped live in Luzon Island, the Philippines [9–11]. We developed an original congenic strain (named B6.Cg-*Pbwg1*) with an approximately 44.1-Mb wild-derived genomic region of mouse chromosome 2 on the B6 genetic background. B6.Cg-*Pbwg1* carries *Pbwg1*, a major body weight QTL among the 24 loci revealed. Genetic analyses using B6.Cg-*Pbwg1* and subcongenic strains created from the descendant of B6.Cg-*Pbwg1* and B6 strains revealed that 12 QTLs for body weight and body composition traits are clustered on the 44.1-Mb wild-derived region [12–15]. Among the 12 loci, two QTLs affecting body weight (named *Pbwg1*.*12*) and the weight of white adipose tissue (WAT) depots (*Pbwg1*.*5*) were recently fine-mapped to a 3.8-Mb region between 61.5 and 65.3 Mb and the neighboring 2.1-Mb region between 59.4 and 61.5 Mb, respectively [16]. At *Pbwg1*.*12*, the wild-derived allele increases body weight despite wild mice having only 60% of the body weight of B6. In contrast, at *Pbwg1*.*5*, the wild-derived allele decreases WAT weight [16] and shows resistance to obesity in mice fed both standard and high-fat diets [17]. In addition, exome sequencing and bioinformatics analysis revealed three nonsynonymous single-nucleotide polymorphisms (nsSNPs) of two putative candidate genes, *Gcg* (glucagon) and *Grb14* (growth factor receptor-bound protein 14), for *Pbwg1*.*12* and 12 nsSNPs of two candidate genes, *Ly75* (lymphocyte antigen 75) and *Itgb6* (integrin beta 6), for *Pbwg1*.*5*, though none of these SNPs was predicted to be harmful to protein functions [16].

The previous studies described above focused on narrowing QTL regions to identify positional and functional candidate genes for *Pbwg1*.*12* and *Pbwg1*.*5* and further to find SNP variants for the candidate genes. In contrast, the aim of the present study was to identify strong candidate genes for two QTLs using an integration approach of transcriptome analysis and the causal inference test (CIT) [18], a statistical method to infer causal relationships between genotype, gene expression and phenotypic difference. By using the integration approach, we successfully identified elevated mRNA expression of the liver *Ly75* gene as a mediator between genotype and obesity-resistant phenotype at *Pbwg1*.*5*.

## Materials and Methods

### Ethics Statement

This study was carried out in accordance with the guidelines for the care and use of laboratory animals of the Graduate School of Bioagricultural Sciences, Nagoya University, Japan. The protocol was approved by the Animal Research Committee of Nagoya University.

### Animals

**Fig 1:**
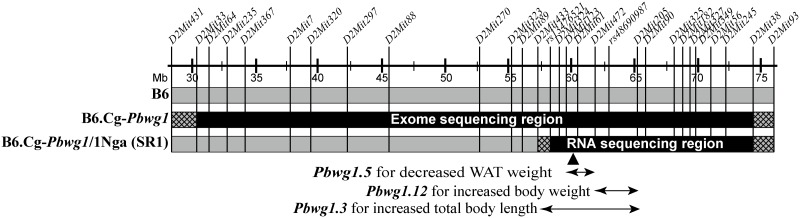
Relative introgressed genomic interval of the B6.Cg-*Pbwg1*/1Nga subcongenic strain (abbreviation SR1). B6.Cg-*Pbwg1*/1Nga was developed from the original B6.Cg-*Pbwg1* congenic strain carrying the *Pbwg1* growth QTL on mouse chromosome 2. The black bar shows the minimum introgressed interval derived from the wild *Mus musculus castaneus* mouse. The genomic region of the black bar in B6.Cg-*Pbwg1* was previously exome-sequenced [16]. The black-bar region of SR1 was RNA-sequenced in this study. The gray bar shows the interval derived from the C57BL/6JJcl (B6) background strain. The hatched bar indicates the interval where recombination occurred. The map positions (Mb) of microsatellite markers (*D2Mit#*) and PCR-RFLP markers (*rs#*) previously developed [16] are shown on the horizontal line. The horizontal double-headed arrows indicate the maximum intervals of body weight and body composition QTLs (*Pbwg1*.*#*) previously defined [16]. The effect of the QTL allele derived from the wild *castaneus* mouse is shown together with the QTL name. The filled triangle indicates the map position of *Ly75*, a strong candidate gene for *Pbwg1*.*5*. WAT, white adipose tissue.

An F_2_ segregating population of 273 mice (138 males and 135 females) was previously established from an intercross between the B6.Cg-*Pbwg1*/1Nga subcongenic strain (called SR1 hereafter) developed from B6.Cg-*Pbwg1* (Fig 1) and its background strain B6 [16]. A set of three F_2_ males was selected from a litter. In the set, three diplotypes for the subcongenic region were segregating. That is, two of the diplotypes were homozygous for either haplotype derived from the wild mouse or from the B6 mouse, and the other was heterozygous for both haplotypes. Finally, five sets of such F_2_ segregating males were selected from four litters. All of the mice were weaned at 3 weeks of age. The mice were given standard chow (CA-1, Clea Japan), containing 5% crude fat and 3.5 kcal/g energy, and tap water *ad libitum*. Detailed husbandry conditions of the mice were described previously [16].

### Growth and Body Composition Traits

All growth and body composition traits for the F_2_ male mice used in this study were previously determined [16]. Briefly, body weights at 1, 3, 6, 10 and 14 weeks of age were recorded. Body weight gains at 1–3 weeks, 3–6 weeks, 6–10 weeks and 10–14 weeks of age were calculated. After overnight fasting, mice were killed on the day after 14 weeks under anesthesia. Total body length (from the tip of the nose to the end of the tail) and tail length (from the anus to the end of the tail) were measured, and head-body length was calculated by subtracting tail length from total body length. Relative head-body and tail lengths were obtained as a percentage of total body length. Blood was obtained by cardiopuncture. The lungs, spleen, liver, kidneys, testes and two sides of inguinal and epididymal WAT depots were weighed. The sum of these two parts of WAT weights was considered as total WAT weight in this study. In mice, the weight of WAT depots has been long and widely used as an indicator of fatness because of a high correlation of the WAT depot weight with total body fat weight [19]. The relative weight of each organ and WAT was obtained as a percentage of body weight at 14 weeks. Parts of the liver and epididymal WAT were stored in RNAlater (Life Technologies, Tokyo) for a few days and subsequently stored at -80°C until total RNA extraction.

### Food Intake

Three male mice of the SR1 strain and four males of the B6 strain at 9–10 weeks of age were housed individually in cages in which the mice had free access to tap water and the powder diet of CA-1. Food intake and body weight were measured every two or three days for 7 days.

### Serum Lipoprotein Analysis

The blood obtained by cardiopuncture was stored at room temperature for approximately 1 hour and overnight at 4°C. Serum was separated by centrifugation at 3,500 rpm for 15 min and stored at -80°C until analysis. Serum lipoproteins were analyzed using gel permeation high-performance liquid chromatography (GP-HPLC) according to the LipoSEARCH^®^ system [20,21] established by Skylight Biotech Inc. (Akita, Japan). Four lipoprotein subclasses, chylomicron (CM), very-low-density lipoprotein (VLDL), low-density lipoprotein (LDL) and high-density lipoprotein (HDL), were calculated for cholesterol (C) and triglycerides (TG). The percentage of each level of C and TG subclasses in total C and total TG levels, respectively, was calculated.

### RNA-sequencing (RNA-seq) Analysis

Total RNA was extracted from the livers of a set of F_2_ segregating males (one individual per diplotype) and from epididymal WATs of three sets of F_2_ segregating males (three individuals per diplotype) using Trizol reagent (Life Technologies, Tokyo) according to the manufacturer's instructions. The concentration and quality of the total RNA were examined by Nanodrop 1000 (Thermo Fisher Scientific, Yokohama, Japan) and Agilent 2100 Bioanalyzer (Agilent Technologies, Tokyo), respectively. For epididymal WAT, the total RNAs of three mice with the same diplotype were pooled because of their low total RNA concentrations. RNA-sequencing (RNA-seq) analysis and subsequent sequence data analyses were outsourced to Hokkaido System Science Co., Ltd (Sapporo, Japan). Briefly, sequence libraries were constructed from the total RNA using a TruSeq RNA sample prep kit (Illumina K.K., Tokyo) according to the manufacturer's instructions. RNA-seq analysis was performed with the next-generation sequencer Illumina Hiseq2000 using a single read of a 101-bp length. The raw sequence data were aligned to the UCSC Mouse Genome Browser GRCm38/mm10 assembly (RefSeq mm10) using TopHat software version 2.0.9 [22]. Values for fragments per kilobase of exon per million mapped fragments (FPKM) were calculated by Cufflinks software version 2.1.1 [22] using the RefSeq mm10 transcriptome. Genes with both FPKM > 0.1 [23] and log_2_(fold change) ≥ 0.58 (or ≤ -0.58) were considered to be differentially expressed in this study. The RNA-seq data were deposited in DDBJ Sequence Read Archive under the accession number of DRA003057.

### Quantitative Real-Time PCR Analysis

Total RNA was extracted from the livers and epididymal WATs of five sets of F_2_ segregating males using Trizol, as described above. Using a PrimeScript^®^ RT reagent Kit with gDNA Eraser (Takara Bio, Otsu, Japan), genomic DNA contamination was removed by the gDNA Eraser enzyme and then cDNA was synthesized from 1 μg of total RNA according to the manufacturer's instructions. Quantitative PCR was performed in a 10.0-μl reaction volume on the StepOnePlus^™^ Real-Time PCR system (Life Technologies, Tokyo) with SYBR^®^ Premix Ex Taq^™^ II (Tli RNaseH Plus) (Takara Bio, Otsu, Japan). Primer sequences for eight candidate genes, *Ly75*, *Pla2r1* (phospholipase A2 receptor 1), *Tank* (TRAF family member-associated Nf-kappa B activator), *Fap* (fibroblast activation protein), *Ifih1* (interferon induced with helicase C domain 1), *Gca* (grancalcin), *Grb14* and *Cobll1* (Cobl-like 1), and two endogenous control genes, *Actb* (actin, beta) and *B2m* (beta-2 microglobulin), were retrieved from the PrimerBank database [24] or designed with Primer Express^®^ version 3.0 (Life Technologies, Tokyo). They were custom-synthesized and are listed in S1 Table. Cycle conditions of quantitative PCR were 95°C for 30 sec and 40 cycles of 95°C for 5 sec and 60°C for 30 sec. All samples were analyzed in triplicate. In the liver, quantitative relative standard curves for the candidate and endogenous genes, with four serial dilution points of the B6 control cDNA (20 ng, 4 ng, 0.8 ng and 0.16 ng), were used to determine mRNA levels. Expression levels of the candidate genes were normalized to a composite level of *Actb* and *B2m* control genes. Dissociation curves, PCR amplification efficiencies and R^2^ values were examined to determine the precision of qPCR. In epididymal WAT, the expression levels of the candidate genes were normalized to that of *Actb* and measured using the 2^−ΔΔCT^ method because of the low concentrations of fat RNA.

### Causal Inference Test (CIT)

**Fig 2:**
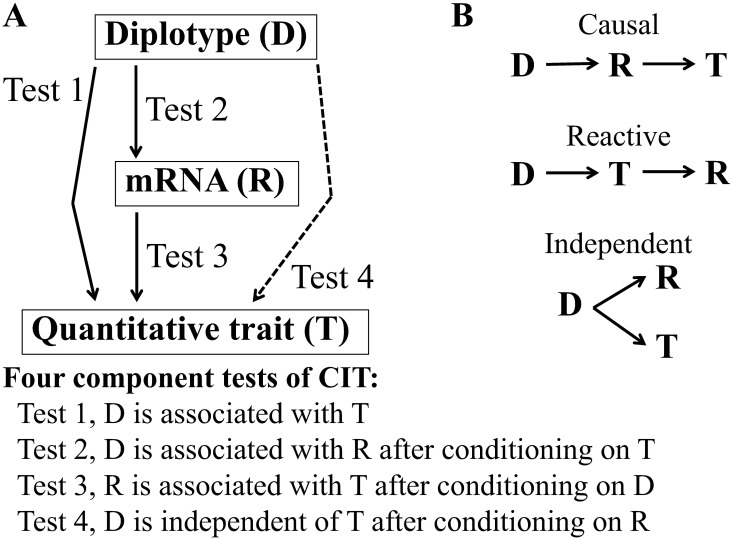
Criteria for causal inference tests (CITs). (A) Four component tests of the CIT [18]. The four component tests assess whether changes in diplotypes (*D*) lead to variation in a quantitative trait (*T*) through changes in mRNA expression (*R*). (B) Possible relationship models estimated from CIT results. Upper, causal model, in which *D* acts on *T* through *R*. Middle, reactive model, in which *R* changes as a result of changes of *T*. Lower, independent model, in which *D* acts on *R* and *T* independently.

The relationships between diplotype (*D*), mRNA expression (*R*) and quantitative trait (*T*) were assessed by causal inference tests (CITs) [18] to determine whether *R* was the mediator between *D*, considered as a cause, and *T*, considered as the phenotypic outcome. Three possible relationship models among these three factors, i.e. causal, reactive and independent relationships, were obtained by the results of CITs (Fig 2). The CIT is composed of four conditional tests that are carried out using the following four linear regression models.

$$T=\beta_{0}+\beta_{1}D+\varepsilon$$ (Model 1)

$$R=\beta_{0}+\beta_{1}D+\beta_{2}T+\varepsilon$$ (Model 2)

$$T=\beta_{0}+\beta_{1}R+\beta_{2}D+\varepsilon$$ (Model 3)

$$T=\beta_{0}+\beta_{1}D+\beta_{2}R+\varepsilon$$ (Model 4)

Here *β*_0_ is the overall mean, *β*_1_ is the effect of the dependent variable of *D* or *R*, *β*_2_ is the effect of the conditioning variable of *T*, *D* or *R*, and *ε* is the random error. For the four CITs, Test 1 determines whether *D* is associated with *T* using Model 1, Test 2 determines whether *D* is associated with *R* after conditioning on *T* using Model 2, Test 3 determines whether *R* is associated with *T* after conditioning on *D* using Model 3, and Test 4 determines whether *D* is independent of *T* after conditioning on *R* using Model 4. To declare a causal relationship, all of the conditional tests must be satisfied.

### Statistical Analysis

The effect of litter on phenotypic values for body weight, body compositions, lipoproteins and gene expression levels obtained in F_2_ males was tested using a linear model of the statistical discovery software JMP version 11.1.1 (SAS Institute, Cary, NC). The litter effect was significant for most of the traits at the nominal 5% level (see S1 Fig for example). Thus, residuals after removing the litter effect were used to perform CITs and to compare phenotypic differences among diplotypes by one-way analysis of variance (ANOVA) followed by Tukey’s honestly significant difference (HSD) *post hoc* test. Food intake was compared between SR1 and B6 strains by Student’s t-test at the nominal 5% level.

## Results

### Trait Analyses

A total of 48 quantitative traits for body weight, growth, body length, organ weight and serum lipoprotein levels were measured in F_2_ male mice with three diplotypes (B/B, B/C and C/C) for the subcongenic region (Fig 1), and all trait values are listed in Table 1. Among the 48 traits, 12 were significantly different among mice with the three diplotypes. Body weights at 10 and 14 weeks of age and body weight gain at 6–10 weeks of age were significantly higher in C/C mice than in B/B mice, despite the fact that the wild mice had only 60% of the body weight of B6 [9]. Percent CM-TG in C/C mice was also significantly higher than in B/B mice. In contrast, absolute and relative weights of epididymal and/or inguinal WATs and testes were significantly lower in C/C mice than in B/B mice. The LDL-C level was also significantly lower in C/C mice than in B/B mice.

**Table 1:** Measurements of 48 quantitative traits for F_2_ mice by diplotype.

	Diplotype				
Trait	B/B	B/C	C/C	*P* value^a^	Differences^b^
No. of mice	5	5	5		
Body weight at 1 week (g)	3.98 ± 0.09	4.11 ± 0.09	4.02 ± 0.09	0.64	NA
Body weight at 3 weeks (g)	7.48 ± 0.26	7.72 ± 0.26	7.55 ± 0.26	0.80	NA
Body weight at 6 weeks (g)	21.16 ± 0.48	21.89 ± 0.48	21.40 ± 0.48	0.56	NA
Body weight at 10 weeks (g)	25.32 ± 0.46	26.85 ± 0.46	27.16 ± 0.46	0.035	C/C ≥ B/C ≥ B/B
Body weight at 14 weeks (g)	27.03 ± 0.45	29.24 ± 0.45	29.39 ± 0.45	0.0052	C/C ≥ B/C > B/B
Weight gain at 1–3 weeks (g)	3.50 ± 0.18	3.61 ± 0.18	3.53 ± 0.18	0.90	NA
Weight gain at 3–6 weeks (g)	13.67 ± 0.34	14.17 ± 0.34	13.85 ± 0.34	0.60	NA
Weight gain at 6–10 weeks (g)	4.16 ± 0.33	4.96 ± 0.33	5.76 ± 0.33	0.016	C/C ≥ B/C ≥ B/B
Weight gain at 10–14 weeks (g)	1.71 ± 0.18	2.38 ± 0.18	2.23 ± 0.18	0.056	NA
Total body length (cm)	17.40 ± 0.05	17.60 ± 0.05	17.55 ± 0.05	0.048	B/C ≥ C/C ≥ B/B
Tail length (cm)	8.29 ± 0.04	8.38 ± 0.04	8.30 ± 0.04	0.28	NA
Head-body length (cm)	9.11 ± 0.04	9.23 ± 0.04	9.26 ± 0.04	0.096	NA
Inguinal WAT weight (g)	0.296 ± 0.015	0.272 ± 0.015	0.250 ± 0.015	0.13	NA
Epididymal WAT weight (g)	0.301 ± 0.013	0.302 ± 0.013	0.251 ± 0.013	0.029	B/C ≥ B/B ≥ C/C
Total WAT weight (g)	0.597 ± 0.027	0.574 ± 0.027	0.502 ± 0.027	0.071	NA
Lungs weight (g)	0.146 ± 0.003	0.154 ± 0.003	0.156 ± 0.003	0.12	NA
Liver weight (g)	1.162 ± 0.031	1.214 ± 0.031	1.234 ± 0.031	0.27	NA
Spleen weight (g)	0.057 ± 0.005	0.071 ± 0.005	0.062 ± 0.005	0.130	NA
Kidneys weight (g)	0.337 ± 0.011	0.375 ± 0.011	0.358 ± 0.011	0.098	NA
Testes weight (g)	0.201 ± 0.005	0.178 ± 0.005	0.187 ± 0.005	0.017	B/B ≥ C/C ≥ B/C
Total-C (mg/g)	59.96 ± 3.13	59.43 ± 3.13	55.73 ± 3.13	0.60	NA
CM-C (mg/g)	0.42 ± 0.06	0.45 ± 0.06	0.59 ± 0.06	0.16	NA
VLDL-C (mg/g)	3.11 ± 0.21	3.33 ± 0.21	2.83 ± 0.21	0.28	NA
LDL-C (mg/g)	5.63 ± 0.25	5.48 ± 0.25	4.67 ± 0.25	0.037	B/B ≥ B/C ≥ C/C
HDL-C (mg/g)	50.79 ± 2.91	50.18 ± 2.91	47.64 ± 2.91	0.73	NA
Total-TG (mg/g)	34.78 ± 3.35	35.10 ± 3.35	35.03 ± 3.35	1.00	NA
CM-TG (mg/g)	3.26 ± 0.76	3.51 ± 0.76	5.44 ± 0.76	0.13	NA
VLDL-TG (mg/g)	16.66 ± 1.88	17.55 ± 1.88	16.74 ± 1.88	0.93	NA
LDL-TG (mg/g)	13.04 ± 0.87	11.97 ± 0.87	10.89 ± 0.87	0.25	NA
HDL-TG (mg/g)	1.82 ± 0.11	2.06 ± 0.11	1.95 ± 0.11	0.32	NA
% Tail length	47.62 ± 0.19	47.59 ± 0.19	47.26 ± 0.19	0.37	NA
% Head-body length	52.38 ± 0.19	52.41 ± 0.19	52.74 ± 0.19	0.37	NA
% Inguinal WAT weight	1.08 ± 0.05	0.93 ± 0.05	0.85 ± 0.05	0.030	B/B ≥ B/C ≥ C/C
% Epididymal WAT weight	1.10 ± 0.05	1.03 ± 0.05	0.85 ± 0.05	0.010	B/B ≥ B/C ≥ C/C
% Total WAT weight	2.18 ± 0.10	1.96 ± 0.10	1.70 ± 0.10	0.019	B/B ≥ B/C ≥ C/C
% Lungs weight	0.54 ± 0.01	0.53 ± 0.01	0.53 ± 0.01	0.36	NA
% Liver weight	4.30 ± 0.10	4.15 ± 0.10	4.19 ± 0.10	0.57	NA
% Spleen weight	0.21 ± 0.01	0.24 ± 0.01	0.21 ± 0.01	0.26	NA
% Kidneys weight	1.24 ± 0.03	1.28 ± 0.03	1.22 ± 0.03	0.28	NA
% Testes weight	0.74 ± 0.01	0.61 ± 0.02	0.64 ± 0.01	0.000069	B/B > C/C ≥ B/C
% CM-C	0.70 ± 0.09	0.79 ± 0.09	1.05 ± 0.09	0.056	NA
% VLDL-C	5.16 ± 0.42	5.75 ± 0.42	5.04 ± 0.42	0.49	NA
% LDL-C	9.40 ± 0.52	9.45 ± 0.52	8.33 ± 0.52	0.27	NA
% HDL-C	84.74 ± 0.95	84.01 ± 0.95	85.57 ± 0.95	0.53	NA
% CM-TG	9.39 ± 1.32	9.89 ± 1.32	15.05 ± 1.32	0.019	C/C > B/C ≥ B/B
% VLDL-TG	46.92 ± 1.65	48.47 ± 1.65	47.38 ± 1.65	0.80	NA
% LDL-TG	38.34 ± 2.05	34.89 ± 2.05	31.75 ± 2.05	0.12	NA
% HDL-TG	5.35 ± 0.59	6.77 ± 0.59	5.81 ± 0.59	0.26	NA

Data are least-squared means and standard errors after adjustment for a litter effect (see Methods).

^a^*P* value for one-way ANOVA.

^b^Significantly different between the three diplotypes at *P* < 0.05 (Tukey's HDS test).

WAT, white adipose tissue; CM-C, chylomicron cholesterol; VLDL-C, very low-density lipoprotein cholesterol; LDL-C, low-density lipoprotein cholesterol; HDL-C, high-density lipoprotein cholesterol; CM-TG, chylomicron triglyceride; VLDL-TG, very low-density lipoprotein triglyceride; LDL-TG, low-density lipoprotein triglyceride; HDL-TG, high-density lipoprotein triglyceride; B, haplotype derived from the C57BL/6JJcl mouse; C, haplotype derived from the wild *M*. *m*. *castaneus* mouse; NA, not applicable.

Food intake was examined for 7 days in males of SR1 subcongenic and B6 strains. Average food intake (± standard error) per day per body weight was 0.156 ± 0.002 g in the SR1 strain and 0.150 ± 0.002 g in the B6 strain. There was no significant strain difference in average food intake.

### RNA-seq Analysis

To screen for genes differentially expressed in the liver and epididymal WAT, RNA-seq was performed on F_2_ male mice that were segregating for the SR1 subcongenic region (Fig 1). Total reads (input reads after trimming) ranging from 52,629,776 bp to 83,051,167 bp were obtained. Approximately 95% of the reads were correctly mapped to RefSeq mm10 in each diplotype by organ (S2 Table).

S3 Table shows expression levels of all genes located in the SR1 subcongenic region between the two microsatellite markers *D2Mit433* and *D2Mit93* on mouse chromosome 2 (see Fig 1). A 5.8-Mb target region harboring *Pbwg1*.*12* and *Pbwg1*.*5* QTLs was physically defined in a previous study [16]. In the target region, only five genes (*Ly75*, *Pla2r1*, *Tank*, *Fap* and *Gca*) were differentially expressed in the liver, and only four genes (*Fap*, *Ifih1*, *Grb14* and *Cobll1*) were differentially expressed in epididymal WAT (Table 2). The expression of *Ly75*, *Tank* and *Fap* was elevated in the liver of mice with the C/C diplotype. *Fap* expression was also increased in WAT. In contrast, *Gca* expression in the liver and *Ifih1 and Grb14* expression in WAT were decreased in the C/C mice. Both *Pla2r1* expression in the liver and *Cobll1* expression in WAT were different in direction of log_2_FC between C/C and B/C diplotypes for unknown reasons.

**Table 2:** Differentially expressed genes in a 5.8-Mb target region of chromosome 2 detected by RNA-seq analysis.

			FPKM			log_2_FC	
Organ	Gene	Position (bp)	C/C	B/C	B/B	C/C to B/B	B/C to B/B
Liver	*Ly75* (lymphocyte antigen 75)	60286265–60383285	1.25	0.41	0.18	2.77	1.16
	*Pla2r1* (phospholipase A2 receptor 1)	60415065–60553308	0.34	0.82	0.59	-0.79	0.47
	*Tank* (TRAF family member-associated Nf-kappa B activator)	61578585–61654183	11.12	8.86	7.14	0.64	0.31
	*Fap* (fibroblast activation protein)	62500715–62575559	0.32	0.20	0.06	2.32	1.64
	*Gca* (grancalcin)	62664298–62695843	0.43	1.02	1.24	-1.52	-0.28
Epididymal WAT	*Fap*	62500715–62575559	4.83	3.65	2.20	1.13	0.73
	*Ifih1* (interferon induced with helicase C domain 1)	62593332–62646255	2.17	2.68	3.70	-0.77	-0.47
	*Grb14* (growth factor receptor-bound protein 14)	64912468–65022766	2.50	4.84	5.84	-1.22	-0.27
	*Cobll1* (Cobl-like 1)	65088183–65240557	0.14	0.33	0.23	-0.67	0.53

The analysis was performed using the liver and epididymal WAT of F_2_ mice with three diplotypes (B/B, B/C and C/C). The target region was physically defined by a previous study [16] (see Fig 1). The positions of the genes are based on the mouse RefSeq mm10. The genes with log_2_FC ≥ 0.58 (≥ 1.5 fold) or ≤ -0.58 (≤ 0.67 fold) are up- or down-regulated.

WAT, white adipose tissue; B, haplotype derived from C57BL/6JJcl; C, haplotype derived from the wild *M*. *m*. *castaneus* mouse; FPKM, fragments per kilobase of exon per million mapped fragments; FC, fold change.

### Quantitative Real-Time PCR Analysis

Although the differential expression of *Tank* in the liver and *Cobll1* in WAT detected by RNA-seq was not validated by quantitative real-time PCR analysis, that of *Ly75*, *Pla2r1*, *Fap*, *Gca*, *Ifih1* and *Grb14* in the liver and/or WAT was validated (Table 3). The C/C mice had significantly higher expression levels of *Ly75* and *Fap* than did the B/B mice. In contrast, the expression levels of *Gca*, *Ifih1* and *Grb14* in the B/B mice were significantly higher than those in the C/C mice. Uniquely, *Pla2r1* expression level was lower in the B/C mice than in the B/B and C/C mice. Furthermore, both the amount and direction of gene expression were very similar in the results of RNA-seq and results of real-time PCR (Tables 2 and 3).

**Table 3:** Validation of differentially expressed genes by quantitative real-time PCR analysis.

		Diplotype				
Organ	Gene	B/B	B/C	C/C	*P* value^b^	Differences^c^
No. of mice^a^		5	5	5		
Liver	*Ly75*	1.00 ± 0.17	1.81 ± 0.17	3.19 ± 0.17	0.0000037	C/C > B/C > B/B
	*Pla2r1*	1.00 ± 0.66	-1.58 ± 0.66	0.58 ± 0.66	0.037	B/B ≥ C/C ≥ B/C
	*Tank*	1.00 ± 2.12	2.24 ± 2.12	-3.24 ± 2.12	0.20	NA
	*Fap*	1.00 ± 0.56	5.89 ± 0.56	8.03 ± 0.56	0.0000042	C/C > B/C > B/B
	*Gca*	1.00 ± 0.03	0.79 ± 0.03	0.34 ± 0.03	0.000000037	B/B ≥ B/C ≥ C/C
Epididymal WAT	*Fap*	1.00 ± 0.06	1.43 ± 0.06	2.11 ± 0.06	0.00000018	C/C > B/C > B/B
	*Ifih1*	1.00 ± 0.31	-0.47 ± 0.31	-0.53 ± 0.31	0.023	B/B > B/C ≥ C/C
	*Grb14*	1.00 ± 0.09	0.73 ± 0.09	0.50 ± 0.09	0.0066	B/B ≥ B/C ≥ C/C
	*Cobll1*	1.00 ± 0.80	-0.85 ± 0.80	-0.15 ± 0.80	0.32	NA

The analysis was performed using F_2_ mice with three diplotypes (B/B, B/C and C/C). The relative expression levels of the B/C and C/C diplotypes to the B/B diplotype are shown as least-squared means and standard errors after adjustment for a litter effect (see Methods).

^a^Three individuals per diplotype were analyzed for *Ifih1* and *Cobll1* genes.

^b^*P* value for one-way ANOVA.

^c^Significantly different between the three diplotypes at *P* < 0.05 (Tukey's HDS test).

WAT, white adipose tissue; B, haplotype derived from C57BL/6JJcl mouse; C, haplotype derived from the wild *M*. *m*. *castaneus* mouse; NA, not applicable.

### CIT Analysis

To identify genes mediating between diplotype and trait, the four component tests of the CIT were carried out as shown in Fig 2. As described earlier, among 48 quantitative traits measured, 12 were significantly associated with diplotype (Table 1), meaning that the 12 traits passed Test 1 of CITs.

**Fig 3:**
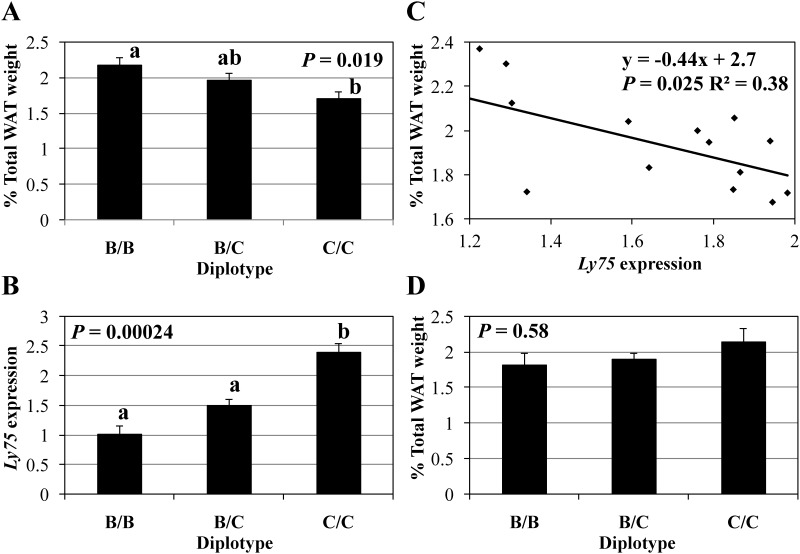
Results of CIT analysis of the percentage of total WAT weight to body weight. (A) Results of Test 1. (B) Results of Test 2. (C) Results of Test 3. (D) Results of Test 4. The total WAT weight is the sum of epididymal and inguinal WAT weights. In (A), (B) and (D), *P* values were obtained from one-way ANOVA of F_2_ mice with three diplotypes, B/B, B/C and C/C (*N* = 5 per diplotype). Data are least-squared means and standard errors. The means with different letters (*a* and *b*) are significantly different between diplotypes at *P* < 0.05 (Tukey’s HDS test). WAT, white adipose tissue.

For *Ly75*, *Pla2r1*, *Fap* and *Gca* genes differentially expressed in the liver, the subsequent three CITs were carried out and the results are summarized in Table 4. Among the four genes, *Ly75*, *Fap* and *Gca* passed Test 2. That is, diplotype was significantly associated with mRNA levels of these three genes after conditioning on each of the 12 traits. However, diplotype was significantly associated with *Pla2r1* expression for only seven traits shown in Table 4. For Test 3 that conditioned on diplotype, *Ly75* expression was significantly associated with percent WAT weight of inguinal and total depots. It was also marginally associated with epididymal WAT weight and percent epididymal WAT weight. *Fap* expression was significantly associated with percent testes weight. *Gca* expression was significantly associated with LDL-C. However, *Pla2r1* expression was not significantly associated with any traits. For Test 4, when conditioning on *Ly75* expression, diplotype was independent of four WAT traits, i.e., epididymal, percent inguinal, percent epididymal and percent total depot weights. When conditioning on *Pla2r1*, *Fap* or *Gca* expression, diplotype was not independent of the corresponding traits. Taken together, only the *Ly75* gene showed a causal relationship with WAT traits. As a typical example of the causal relationship, the results of all four tests for percent total WAT weight are shown in Fig 3.

**Table 4:** Results for Tests 2–4 of CIT analysis of four genes differentially expressed in the liver.

		*P* value			
Trait	Gene	Test 2	Test 3	Test 4	Relationship model^a^
Body weight at 10 weeks	*Ly75*	0.000083	0.60	0.36	
	*Pla2r1*	0.071	NA	NA	
	*Fap*	0.00021	0.60	0.80	
	*Gca*	0.00000089	0.88	0.27	
Body weight at 14 weeks	*Ly75*	0.00013	0.67	0.098	
	*Pla2r1*	0.075	NA	NA	
	*Fap*	0.00044	1.00	0.30	
	*Gca*	0.0000013	0.82	0.068	
Weight gain at 6–10 weeks	*Ly75*	0.00040	0.13	0.83	
	*Pla2r1*	0.037	0.47	0.017	Independent
	*Fap*	0.00040	0.19	0.74	
	*Gca*	0.0000042	0.14	0.60	
Total body length	*Ly75*	0.000025	0.72	0.14	
	*Pla2r1*	0.018	0.16	0.023	Independent
	*Fap*	0.000015	0.22	0.076	
	*Gca*	0.00000031	0.63	0.13	
Epididymal WAT weight	*Ly75*	0.000087	0.057^b^	0.32	Causal^b^
	*Pla2r1*	0.058	NA	NA	
	*Fap*	0.000025	0.088	0.083	
	*Gca*	0.00000079	0.36	0.20	
Testes weight	*Ly75*	0.000023	0.38	0.025	Independent
	*Pla2r1*	0.28	NA	NA	
	*Fap*	0.00013	0.088	0.10	
	*Gca*	0.00000022	0.30	0.020	Independent
LDL-C	*Ly75*	0.000029	0.27	0.13	
	*Pla2r1*	0.025	0.23	0.025	Independent
	*Fap*	0.000062	0.92	0.26	
	*Gca*	0.0000000016	0.00071	0.00050	
% Testes weight	*Ly75*	0.000089	0.44	0.000631	Independent
	*Pla2r1*	0.073	NA	NA	
	*Fap*	0.0021	0.035	0.011	
	*Gca*	0.00000051	0.20	0.00032	Independent
% Inguinal WAT weight	*Ly75*	0.000055	0.015	0.21	Causal
	*Pla2r1*	0.013	0.10	0.011	Independent
	*Fap*	0.00027	0.46	0.93	
	*Gca*	0.00000069	0.35	0.18	
% Epididymal WAT weight	*Ly75*	0.00054	0.058^b^	0.78	Causal^b^
	*Pla2r1*	0.043	0.47	0.014	Independent
	*Fap*	0.00015	0.10	0.20	
	*Gca*	0.0000027	0.41	0.27	
% Total WAT weight	*Ly75*	0.00024	0.025	0.58	Causal
	*Pla2r1*	0.025	0.22	0.014	Independent
	*Fap*	0.00030	0.24	0.66	
	*Gca*	0.0000015	0.37	0.25	
% CM-TG	*Ly75*	0.000093	0.62	0.23	
	*Pla2r1*	0.049	0.35	0.026	Independent
	*Fap*	0.000035	0.50	0.076	
	*Gca*	0.0000041	0.69	0.76	

^a^See Fig 1 for relationship models.

^b^*P* values for Test 3 are marginal.

CIT, causal inference test; WAT, white adipose tissue; LDL-C, low-density lipoprotein of cholesterol; CM-TG, chylomicron of triglyceride; NA, not applicable.

In addition, both *Ly75* and *Gca* genes showed independent relationships with testes weight traits. The *Pla21* gene also showed independent relationships with body weight gain at 6–10 weeks, total body length, WAT and lipoprotein traits, though their *P* values for Tests 2 and 4 were not so low (Table 4). None of the genes differentially expressed in the liver had causal relationships with body weight and growth traits.

Although *Fap*, *Ifih1* and *Grb14* gene expression in epididymal WAT passed Test 2, only *Ifih1* passed the subsequent two tests (Table 5). *Ifih1* finally showed a reactive relationship with body weight gain at 6–10 weeks and an independent relationship with percent CM-TG. However, none of the genes differentially expressed in epididymal WAT showed causal relationships with body weight, growth, WAT and lipoptotein traits.

**Table 5:** Results for Tests 2–4 of CIT analysis of the *Ifih1* gene differentially expressed in epididymal WAT.

	*P* value			
Trait	Test 2	Test 3	Test 4	Relationship model^a^
Body weight at 10 weeks	0.14	NA	NA	
Body weight at 14 weeks	0.062	NA	NA	
Weight gain at 6–10 weeks	0.0027	0.015	0.0031	Reactive
Total body length	0.10	NA	NA	
Epididymal WAT weight	0.068	NA	NA	
Testes weight	0.23	NA	NA	
LDL-C	0.047	0.66	0.54	
% Testes weight	0.14	NA	NA	
% Inguinal WAT weight	0.055	NA	NA	
% Epididymal WAT weight	0.11	NA	NA	
% Total WAT weight	0.076	NA	NA	
% CM-TG	0.024	0.29	0.030	Independent

^a^See Fig 1 for relationship models.

CIT, causal inference test; WAT, white adipose tissue; LDL-C, low-density lipoprotein of cholesterol; CM-TG, chylomicron of triglyceride; NA, not applicable.

## Discussion

The integration approach of gene expression and the CIT clearly demonstrated that the *Ly75* gene is the mediator between diplotype and WAT weight, indicating that *Ly75* is a strong candidate gene for the *Pbwg1*.*5* QTL discovered from an untapped natural resource of wild *M*. *m*. *castaneus* mice in the Philippines. In contrast, the integration approach failed to find candidate genes for the *Pbwg1*.*12* QTL for body weight because none of the genes differentially expressed in the liver and epididymal WAT was causally associated with body weight and growth traits.

*Ly75* encodes DEC-205 (dendritic and epithelial cells, 205 kDa), which is also known as CD205 (cluster of differentiation 205). DEC-205 is an integral membrane protein homologous to the macrophage mannose receptor, and it is a novel endocytic receptor used by dendritic cells and thymic epithelial cells to direct captured antigens from the extracellular space to a specialized antigen-processing compartment [25]. Knockout mice for *Ly75* have abnormalities in CD8-positive T cell morphology and cytotoxic T cell physiology [26], though the direct effect of *Ly75* on WAT and related traits has not yet been investigated. Hence, it is not precisely known how *Ly75* expression in the liver regulates WAT weight.

A previous microarray study [27] revealed 259 genes differentially expressed in the liver between SM/J and LG/J strains of mice fed a high-fat diet. The SM/J strain is more responsive than the LG/J strain for many obesity and diabetes traits. Most of the differentially expressed genes are associated with immune function, and 62 genes are located within intervals of QTLs previously mapped for obesity, diabetes and related traits [27]. Since high-fat diets are known to trigger an immune response through inflammation in many organs and tissues such as the liver and adipose tissue [28,29], genes associated with immune function can become candidate genes for obesity and diabetes QTLs. In fact, in humans, the SNP associated with *LY75* gene expression has been reported to be relevant to type 2 diabetes mellitus [30]. In chickens, *LY75* has been defined as a candidate gene for an adiposity QTL [31]. These facts thus reinforce the mouse *Ly75* gene as a putative QTG for the *Pbwg1*.*5* QTL with a preventive effect on obesity when mice are fed both low-fat standard and high-fat diets [17].

Previous exome sequencing analysis of the wild-derived congenic region on mouse chromosome 2 revealed nine nsSNPs, leading to amino-acid changes, in *Ly75* exons, but none of the nsSNPs was predicted to be harmful to protein function [16]. We thus consider that some kinds of DNA changes in the promoter, enhancer or intronic region of *Ly75* or epigenetic changes cause the expression difference in the liver *Ly75* gene between wild and B6 mice. As shown in S4 Table, many genes associated with immune functions were differentially expressed in the liver of the F_2_ mice used in this study. The differentially expressed genes were located on all chromosomes excluding the *Pbwg1*.*5* QTL region of chromosome 2 and Y chromosome. Some of the genes associated with immune functions might be downstream genes for which expression is affected by *Ly75* expression.

A quantitative complementation test, or a QTL-knockout interaction test, is often used to determine whether a candidate gene is a true QTG in rodents [32–34]. In the test, two experimental strains with different QTL alleles on different genetic backgrounds are each crossed to two tester strains, a knockout strain and its background strain with a wild-type allele. Trait values for F_1_ hybrids produced from the four crosses are measured. An interaction effect between the knockout allele and the QTL allele is examined on the trait. If no interaction effect is obtained, then it is interpreted as evidence that the candidate gene is not a QTG. If the interaction effect is statistically significant, it is concluded that the candidate gene is a QTG, but the possibility that the interaction effect is caused by epistatic interaction between the candidate gene and other QTLs on different chromosomal regions cannot be entirely ruled out because the genetic backgrounds of the two experimental strains are different. To overcome this problem, a congenic strain carrying the QTL and its background strain with a wild-type QTL allele are used as experimental strains to perform the quantitative complementation test in a common genetic background [32,35]. Quantitative complementation tests using SM/J and LG/J mice revealed that the *Capn10* (calpain-10) gene is a QTG for *Adip1*, an obesity QTL identified in a set of recombinant inbred strains between SM/J and LG/J, because highly significant interaction effects between *Capn10* knockout and *Adip1* genotypes were obtained for body weight, weights of fat pads, liver and heart, and serum leptin level [34]. Quantitative complementation tests in a common mouse genetic background suggested that the *Pappa2* (pregnancy-associated plasma protein A2) gene is a QTG for a QTL with a small general effect on body size (tail length, bone length and body weight) found in a cross between DBA/2J and C57BL/6J mice; however, an interaction between *Pappa2* and QTL genotypes was only significant for tail length and body weight at 3 weeks of age, whereas it was not significant for lengths of the skull and long bones and body weight at 6 weeks and 10 weeks [36]. Hence, even if a gene that has been knockouted is a QTG for a QTL with small phenotypic effects, the results of quantitative complementation tests would not always reach statistical significance. Another approach is to use a simple transgenic overexpression of a candidate gene, which has recently been proved efficient for positional cloning of a tail suspension QTL [5] and an adiposity QTL [7] in mice. We are now planning to perform a quantitative complementation test and/or a simple overexpression experiment using the *Ly75* cDNA of *M*. *m*. *castaneus* in order to finally confirm the causality between *Ly75* and *Pbwg1*.*5*.

## Conclusions

The results of this study provided the first statistical evidence that *Ly75* expression mediated between diplotype and WAT in mice, suggesting that *Ly75* is a putative QTG for the obesity-resistant *Pbwg1*.*5* QTL discovered from the wild *M*. *m*. *castaneus* mouse. This finding provides a novel insight into a better understanding of the genetic basis for prevention of obesity.

## Supporting Information

S1 Fig Effect of litter on total WAT weight. Data are means and standard errors. The effect of four litters, A (*N* = 6), B (*N* = 3), C (*N* = 3) and D (*N* = 3), was highly significant at *P* = 0.000010 (one-way ANOVA). The means with different letters (a, b and c) are significantly different between litters at *P* < 0.05 (Tukey’s HDS test).
(TIF)

S1 Table List of qPCR primers used in this study. ^a^F, forward primer; R, reverse primer. ^b^PrimerBank database [24]; NA, not aplicable but designed with Primer Express^®^ version 3.0 (Life Technologies, Tokyo).
(XLSX)

S2 Table Summary of RNA-seq analysis of the liver and epididymal WAT in F_2_ mice. Data are shown in bp. ^a^Three diplotypes, B/B, B/C and C/C, were analyzed. WAT, white adipose tissue; B, haplotype derived from C57BL/6JJcl; C, haplotype derived from the wild *M*. *m*. *castaneus* mouse.
(XLSX)

S3 Table Expression of all genes in the SR1 subcongenic region detected by RNA-seq analysis using the liver and epididymal WAT of F_2_ mice with three diplotypes (B/B, B/C and C/C). Genes with an expression level with FPKM > 0.1 [23] are focused on. ^a^UP in parenthesis denotes an up-regulated gene with log_2_FC ≥ 1.0, DOWN indicates a down-regulated gene with log_2_FC ≤ -1.0, and DIS indicates a gene discrepant in direction of differentially expressed levels between the liver and WAT. ^b^Based on the mouse RefSeq mm10. ^c^R denotes regions where recombination occurred, and T denotes a target region physically defined by a previous study [16]. B, haplotype derived from C57BL/6JJcl; C, haplotype derived from the wild M. *m*. *castaneus* mouse; FPKM, fragments per kilobase of exon per million mapped fragments; FC, fold change; NA, not applicable.
(XLSX)

S4 Table Differentially expressed genes on all chromosomes detected by RNA-seq analysis using the livers of F_2_ mice with three diplotypes (B/B, B/C and C/C). The differentially expressed genes with FPKM > 0.1 [23] are listed at the threshold level of log_2_FC ≥ 0.58 (≥ 1.5 fold) or ≤ -0.58 (≤ 0.67 fold). The positions of the genes are based on the mouse RefSeq mm10. The genes associated with immune functions (see MGD [2]) are shown in bold font. ^a^Q denotes the genomic region harboring two QTLs for body weight (*Pbwg1*.*12*) and resistance to obesity (*Pbwg1*.*5*). B, haplotype derived from C57BL/6JJcl; C, haplotype derived from the wild *M*. *m*. *castaneus* mouse; FPKM, fragments per kilobase of exon per million mapped fragments; FC, fold change.
(XLSX)
