# Correction: Identification of a Putative Quantitative Trait Gene for Resistance to Obesity in Mice Using Transcriptome Analysis and Causal Inference Tests

**DOI:** 10.1371/journal.pone.0175006

**Published:** 2017-03-27

**Authors:** Akira Ishikawa

There are errors in the second and last sentences of the last paragraph in the Results section. The correct second sentence should read: *Ifih1* finally showed an independent relationship with percent CM-TG. In the last sentence, “lipoptotein” should be “lipoprotein.”

There are errors in the second and last sentences of the footnote in S3 Table. In the second sentence, log_2_FC ≥ “1.0” should be log_2_FC ≥ “0.58 (≥ 1.5-fold)”, and log_2_FC ≤ “-1.0” should be log_2_FC ≤ “-0.58 (≤ 0.67-fold).” In the last sentence, “M” of *M*. *m*. *castaneus* should be indicated in italics.

There is an error in the Relationship model column of Table 5. “Reactive” should be deleted. Please see the corrected Table 5 here.

**Table 5:** Results for Tests 2–4 of CIT analysis of the *Ifih1* gene differentially expressed in epididymal WAT.

	*P* value			
Trait	Test 2	Test 3	Test 4	Relationship model^a^
Body weight at 10 weeks	0.14	NA	NA	
Body weight at 14 weeks	0.062	NA	NA	
Weight gain at 6–10 weeks	0.0027	0.015	0.0031	
Total body length	0.10	NA	NA	
Epididymal WAT weight	0.068	NA	NA	
Testes weight	0.23	NA	NA	
LDL-C	0.047	0.66	0.54	
% Testes weight	0.14	NA	NA	
% Inguinal WAT weight	0.055	NA	NA	
% Epididymal WAT weight	0.11	NA	NA	
% Total WAT weight	0.076	NA	NA	
% CM-TG	0.024	0.29	0.030	Independent

^a^See Fig 1 for relationship models. CIT, causal inference test; WAT, white adipose tissue; LDL-C, low-density lipoprotein of cholesterol; CM-TG, chylomicron of triglyceride; NA, not applicable.
