# Supplementary figures and images for: Identification of a Putative Quantitative Trait Gene for Resistance to Obesity in Mice Using Transcriptome Analysis and Causal Inference Tests
Source: PLoS One. 2017 Jan 23;12(1):e0170652. doi: 10.1371/journal.pone.0170652 (PMC5256930; doi:10.1371/journal.pone.0170652)

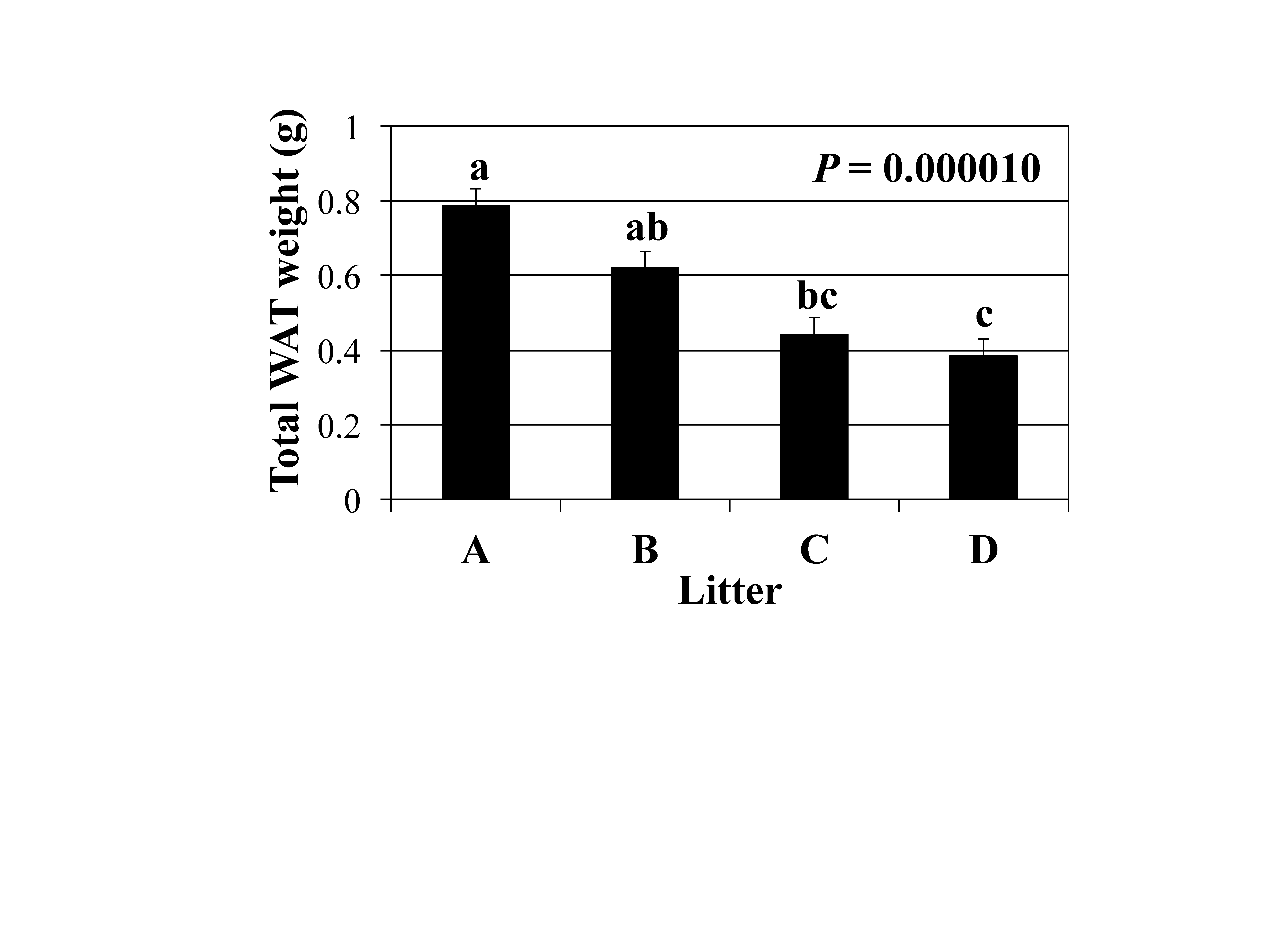

Supplement: S1 Fig — Data are means and standard errors. The effect of four litters, A (N = 6), B (N = 3), C (N = 3) and D (N = 3), was highly significant at P = 0.000010 (one-way ANOVA). The means with different letters (a, b and c) are significantly different between litters at P < 0.05 (Tukey’s HDS test). (TIF) [file pone.0170652.s001.tif]
